# A Comparison Between Somatosensory Evoked Potentials and Spine MRI in the Diagnosis of Non-compressive Myelopathy: Which Is More Accurate?

**DOI:** 10.7759/cureus.38051

**Published:** 2023-04-24

**Authors:** Haneen J Mohammed, Mazin M Hammady, Farah N Abbas

**Affiliations:** 1 Department of Physiology, Basrah Health Directorate, Basrah, IRQ; 2 Department of Internal Medicine, College of Medicine, University of Basrah, Basrah, IRQ; 3 Department of Physiology, College of Medicine, University of Babylon, Babylon, IRQ

**Keywords:** diagnosis, accuracy, non-compressive myelopathy, spine mri, sseps

## Abstract

Introduction: Non-compressive myelopathy is a neurological disorder due to pathological processes affecting the spinal cord in the absence of clinical and radiological evidence of spinal cord compression. Two commonly used diagnostic tools for non-compressive myelopathy are somatosensory evoked potentials (SSEPs) and magnetic resonance imaging (MRI). SSEPs are a neurophysiological tool used to assess the functional integrity of the spinal cord. MRI, on the other hand, is the mainstay imaging modality used for identifying compressive lesions and other structural abnormalities in the spinal cord. The aim of this study was to test the diagnostic accuracy of SSEPs versus spine MRI in the diagnosis and assessment of the severity of non-compressive myelopathy using the Modified Japanese Orthopaedic Association (mJOA) clinical severity score.

Methods: Our study included 63 subjects. Whole spine MRI and SSEPs (median and tibial SSEP bilaterally) were done for all subjects; their results were compared according to their relation to the mJOA score and classified into mild, moderate, and severe. The control group was examined to establish normative data for SSEP results and compared with cases. Blood investigations such as complete blood count, thyroid function test, A1C, HIV tests, venereal disease research laboratory test, erythrocyte sedimentation rate, C-reactive protein, and antinuclear antibody tests were done. Blood tests for vitamin B12 levels were done for patients who were suspected of sub-acute combined degeneration of the spinal cord; cerebrospinal fluid (CSF) analysis was done for patients suspected of multiple sclerosis (MS), acute transverse myelitis (ATM), or other inflammatory/infectious diseases. CSF was analyzed for cell count, cytology, protein, and oligoclonal bands (if indicated).

Results: No mild categories were registered in this study; 30% of patients were moderate and 70% were severe. Causes for non-compressive myelopathy in this study were hereditary degenerative ataxias in 12 (38.71%), ATM in 8 (25.81%), and MS in 5 (16.13%); other causes included vitamin B12 deficiency in 2 (6.45%), ischemia in 2 (6.45%), and an unknown cause in 2 (6.45%). SSEPs showed abnormal results in all patients (31; 100%) whereas MRI showed abnormality in only seven patients (22.6%). SSEP sensitivity for detecting severe cases was around 63.6% while that for MRI was 27.3%.

Conclusion: The study concluded that SSEPs were more reliable for the detection of non-compressive myelopathies rather than MRI and correlated better with clinical severity. Performing SSEPs is recommended for all patients with non-compressive myelopathy, especially those with negative imaging.

## Introduction

Non-compressive myelopathy is a term describing any neurological deficit resulting from a pathological process affecting the spinal cord in the absence of clinical and radiological evidence of spinal cord compression. Myelopathy includes different etiologies with heterogenic pathogenesis but nearly similar clinical presentation [1]. The non-compressive causes are mainly vascular (ischemia), inflammatory (multiple sclerosis, or MS, and transverse myelitis), and others (degenerative, metabolic/toxic, radiation, and paraneoplastic) [2,3]. Imaging plays a crucial role in refining the differential diagnosis. Magnetic resonance imaging (MRI) is now the mainstay in the evaluation of myelopathy because of its superior soft-tissue resolution and multiplanar capability, making it ideal for the evaluation of the spinal canal and its contents as well as the surrounding bony and soft-tissue structures in both compressive and non-compressive myelopathy [4,5]. Sagittal and axial T1- and T2-weighted and short-tau inversion-recovery (STIR) MRI sequences typically are used in the diagnosis of myelopathy and may be supplemented by fat-suppressed, gradient-echo, diffusion-weighted, and contrast-enhanced sequences. Conventional MRI is frequently incapable of distinguishing the ongoing pathology in normal-appearing white matter (NAWM), despite known disease processes as described with histopathological correlation. Some spinal cord syndromes can present with normal or near-normal MRI findings; this condition occurred in about one-fifth of cases of myelopathy referred to a UK neurosciences center [6,7].

Somatosensory evoked potentials (SSEPs) have been routinely used over the years to evaluate the somatosensory pathway along the spinal cord up to the cortical level, and thereby supplement the diagnostic process when the history, neurologic examination, and imaging are not fully conclusive [8]. The stimulation sites that are frequently used to elicit SSEP waves are the median nerve in the wrist, the common peroneal nerve in the knee, and the tibial nerve in the ankle [9]. There are several functional disability measures that can be used to assess the clinical severity of myelopathies such as the Cooper Myelopathy Scale, Nurick Scale, European Myelopathy Score (EMS), Modified Japanese Orthopaedic Association (mJOA) score, Myelopathy Disability Index (MDI) and other scores. These scores involve the assessment of the motor function of upper and lower limbs, sensory level, and sphincter control with a grading system; the lower the grade, the more severe the deficit [10]. The mJOA score is the score most often used in the published literature. The Nurick score mainly emphasizes gait dysfunction where mild myelopathy is defined as an mJOA score ranging from 15 to 17, moderate myelopathy as 12 to 14, and severe myelopathy as an mJOA score from 0 to 11 [11,12].

This study was conducted to test the diagnostic accuracy of SSEPs versus spine MRI in the diagnosis and assessment of the severity of non-compressive myelopathy using the mJOA clinical severity score.

## Materials and methods

This study involved 63 subjects: 32 controls (mean age 29 ± 11.9 years) and 31 patients (mean age 30.26 ± 12.94 years). Patients were classified according to the mJOA scoring system into mild, moderate, and severe. Ethical clearance was taken from the Basrah Health Directorate (IEC/PHYSIO/121).

Whole spine MRI and SSEPs (median and tibial SSEP bilaterally) were done for all subjects and their results were compared according to their relation to the mJOA score. The median and tibial nerve SSEPs were analyzed. The tibial nerve was stimulated at a rate of 2.9 Hz or less when it was stimulated at the ankle, whereas the median nerve was stimulated at a rate of 4.9 Hz when it was stimulated at the wrist. The antidromic sensory nerve action potential was used to help determine the stimulus intensity for the median nerve SSEPs. The intensity of the stimulus for the tibial nerve SSEPs was increased to a level that was twice as intense as the threshold for radiating paresthesia toward the toes. Electroencephalogram (EEG) disc electrodes were placed at a number of different locations, including the Erb's point ipsilateral to the stimulation (EPi), the spinous process of the sixth cervical vertebra (C6S), the contralateral centroparietal electrode (CPc) and a non-cephalic (NC) reference over the contralateral shoulder for median nerve SSEPs, and the contralateral iliac crest (ICc), spinous processes of the first lumbar and second cervical vertebrae. The evoked potentials were amplified, and the frequency range was changed to be between 5 and 1500 or 2000 Hz (-3 db). The median nerve SSEPs received an average of 1200-2500 responses, while the tibial nerve SSEPs received an average of 200-800 responses. A signal processor DP1100 (NEC San-ei Co., Tokyo) or a Neuropack 2000 (Nihon Kohden Co., Tokyo) was utilized in order to superimpose two or more averages from separate electromyography (EMG) machines. The SSEPs of the median nerve and the tibial nerve were examined bilaterally in the subjects. When this occurred, the SSEPs that were recorded after stimulation on the side of the patient with more severe clinical deficits were the ones that were analyzed.

The MRI films were evaluated objectively and independently by one of the authors, who was blinded to the clinical data. Based on sagittal and axial T2-weighted images, the findings at each intervertebral disc level were graded as follows: grade 0 indicated that there was no impingement on the spinal cord; grade 1 indicated that there was some impingement, but the deformity of the spinal cord was absent or mild; grade 2 indicated that there was an evident deformity of the spinal cord with an obviously reduced cross-sectional area; and grade 3 indicated that there was a grade 2 deformity in addition to a high-signal-intensity area within the spinal cord on the T2-weighted image. A positive MRI sign supporting cervical spondylotic myelopathy (CSM) was considered to be changes of grade 2 or 3 occurring at a minimum of one intervertebral level.

To rule out peripheral neuropathy (not connected to myelopathy) that can alter SSEP results or the diagnosis, nerve conduction study (NCS) and EMG tests were conducted (myeloneuropathies influencing N9 or popliteal fossa [PF] latencies were excluded). The control group was examined to establish normative data for SSEP results and compared with cases. Blood investigations such as complete blood count, thyroid function test, A1C, HIV test, venereal disease research laboratory (VDRL) test, tests for erythrocyte sedimentation rate, C-reactive protein, and antinuclear antibody tests were done. Blood tests were conducted for vitamin B12 levels in patients who were suspected of sub-acute combined degeneration of the spinal cord; cerebrospinal fluid (CSF) analysis was done for patients suspected of multiple sclerosis, acute transverse myelitis (ATM), or other inflammatory/infectious diseases. CSF was analyzed for cell count, cytology, protein, and oligoclonal bands (if indicated).

## Results

Clinical characteristics of patients included in the study are given in Table 1. No mild myelopathy was registered; 30% had moderate severity myelopathy and 70% had a severe form according to mJOA clinical assessment. Regarding the clinical site of myelopathy, 18 (58%) patients had diffuse myelopathy involving most of the spinal cord segments, mainly attributed to patients with hereditary degenerative ataxias; 9 (29%) patients had dorsal myelopathy mainly related to ATM and MS. The most common cause was hereditary degenerative ataxias (12, 37.1%), followed by ATM (8, 25.81%).

**Table 1:** Characteristics of non-compressive myelopathy patients n, number of patients

Patient characteristics		n = 31
Age (years)		30.26 ± 12.94
Sex	Male	15 (48.4%)
	Female	16 (51.6%)
Clinical site of compression	Cervical	1 (3%)
	Dorsal	9 (29%)
	Lumbar	3 (10%)
	Multiple segments	18 (58.0%)

Since the tibial SSEPs are the longest pathway for signal conduction along the spinal cord, they are more reliable for detecting a spinal cord abnormality. The SSEP conduction delay is divided into two categories: (1) prolonged conduction and (2) absent or delayed poor waveform (Ab/PWF), with the median of P37 latency being around 65 ms (Table 2).

**Table 2:** Comparison of P37 latencies on tibial SSEPs for prolonged and Ab/PWF n, number of patients; SSEP, somatosensory evoked potential; Ab/PWF, absent/poor waveform *Significant

SSEP conduction type		P37 latency
Prolonged	n	17
	Mean	55.17
	Median	51.50
	SD	7.51
	Minimum	47.70
	Maximum	67.20
Ab/PWF	N	14
	Mean	56.03
	Median	65.20
	SD	23.89
	Minimum	0.00
	Maximum	69.00
p-value*	0.032	

When comparing mJOA scores with SSEP results, all patients with moderate myelopathy (100%) had prolonged conduction and none of them had Ab/PWF, while 63.6% of severe cases had Ab/PWF on SSEPs (Table 3).

**Table 3:** Relationship between mJOA scores in patients with non-compressive myelopathy and SSEP conduction severity mJOA score, Modified Japanese Orthopaedic Association score; SSEP, somatosensory evoked potential; Ab/PWF, absent/poor waveform

Variable			mJOA scores		Total
			Moderate	Severe	
SSEP conduction severity	Prolonged	Count	9	8	17
		Column %	100%	36.4%	54.8%
	Ab/PWF	Count	0	14	14
		Column %	0.0%	63.6%	45.2%
Total		Count	9	22	31
		% of total	29.0%	71.0%	100%

**Figure 1:**
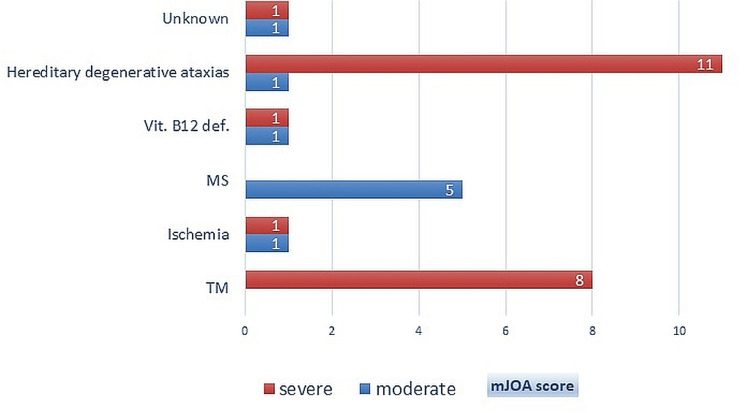
Distribution of causes of non-compressive myelopathy according to the mJOA scale mJOA scale, Modified Japanese Orthopaedic Association scale, MS, multiple sclerosis; TM, transverse myelitis

In our study, all patients with ATM and most of the patients with hereditary degenerative ataxias had severe myelopathy while all MS patients had moderate mJOA scores (Figure 1).

In this study, SSEPs showed abnormality in all patients (either median or tibial or both) while spine MRI showed white matter abnormality (WMA) in only seven patients and all other patients had normal-appearing white matter as displayed in Table 4.

**Table 4:** Distribution of mJOA scores for patients with non-compressive myelopathy according to spine MRI abnormality (normal and abnormal) mJOA score, Modified Japanese Orthopaedic Association score; MRI, magnetic resonance imaging

Variable			mJOA standard		Total
			Moderate	Severe	
MRI abnormality	Normal	Count	8	16	24
		Column %	88.9%	72.7%	77.4%
	Abnormal	Count	1	6	7
		Column %	11.1%	27.3%	22.6%
Total		Count	9	22	31
		% of total	29.0%	71.0%	100%

**Figure 2:**
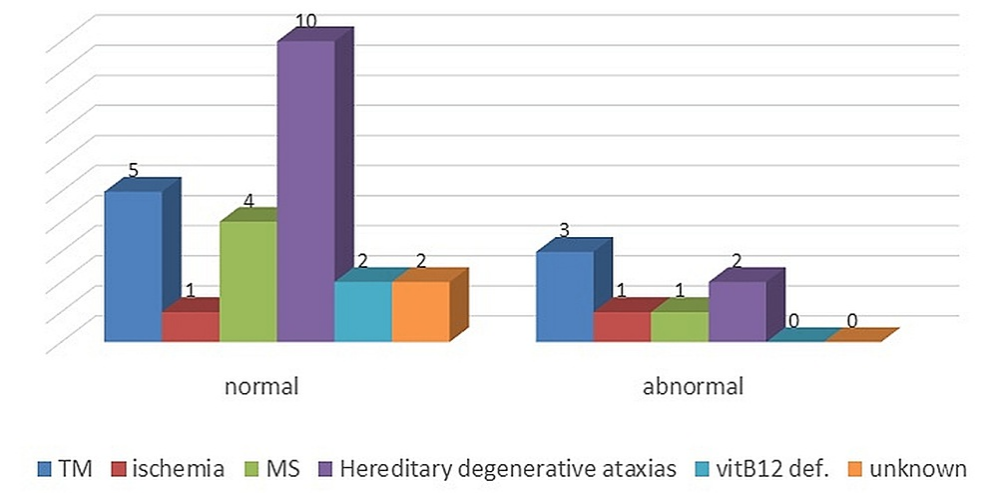
Spine MRI with white matter abnormality in non-compressive myelopathy according to causes MS, multiple sclerosis; TM, transverse myelitis

Spine MRI showed WMA in three cases of TM while abnormal in one case of hereditary degenerative ataxias (Figure 2).

In hereditary degenerative ataxias, conduction delay is diffuse over all spinal segments along with cortical and subcortical delayed evoked responses denoted by the prolonged interpeak latency (IPL) P31-P37. Table 5 shows delayed tibial SSEPs (latencies and IPL).

**Table 5:** Tibial SSEPs in patients with hereditary degenerative ataxias SSEPs, somatosensory evoked potentials; IPL, interpeak latency

Latency and IPL	Upper limit (mean ± SD)	Lower limit (mean ± SD)
N22	30.4 ± 1.41	23.16 ± 1.68
P31	43.26 ± 1.96	31.32 ± 1.45
P37	60.92 ± 2.93	38.58 ± 1.49
IPL N22-P37	30.50 ± 3.03	15.41 ± 1.87
IPL N22-P31	12.85 ± 1.51	8.16 ± 1.37

All patients with ATM had severe mJOA myelopathy scale scores and only three patients had a spine MRI finding of WMA. Dorsal spine segments were the segments most affected (in four patients) followed by cervical spine segments (in only two patients). Table 6 illustrates tibial SSEP latencies and IPL in ATM that showed delayed P31 and IPL N22-P31, but normal IPL P31-P37.

**Table 6:** Tibial SSEPs in patients with transverse myelitis SSEPs, somatosensory evoked potentials; IPL, interpeak latency

Latency and IPL	Upper limit (mean ± SD)	Lower limit (mean ± SD)
N22	35.84 ± 22.31	23.16 ± 1.68
P31	56.76 ± 16.2	31.32 ± 1.45
P37	60.21 ± 11.2	38.58 ± 1.49
IPL N22-P31	28.1 ± 21.22	8.16 ± 1.37

## Discussion

Our study involved 63 subjects in which hereditary degenerative ataxias were the most common cause for non-compressive myelopathy accounting for 12 (38.71%), with their age ranging from 18 to 28 years; some of the patients were related. Prabhakar et al. studied 52 patients with non-compressive myelopathy, in which ATM was the commonest cause (in 31) followed by vitamin B12 deficiency (in 8) [13]. Due to the very small sample sizes used in both studies to calculate the prevalence rates of the causes, it is possible that the differences in the underlying etiologies are the result of a variety of factors, including the method used to collect the cases. The SSEP sensitivity for detecting severe cases was around 63.6% while MRI sensitivity was 27.3%. When clinical signs alone were used as the gold standard, the sensitivity of median SSEPs was 90% whereas that of MRI was 88% in patients with cervical spondylotic myelopathy as documented by Nakai et al. [14]. All patients with hereditary degenerative ataxias had ataxia and slurred speech, and 10 patients had sensory neuropathy by NCS (but did not affect N9 or PF latencies on SSEPs). MRI was abnormal in only two patients showing cerebellar atrophy and white matter atrophy of the spinal cord while other patients had normal spine and brain MRI. Median and tibial SSEPs were abnormal in all patients in our study in comparison with the control group with prolonged latencies and IPL, especially IPL 31-37 that denoted cortical and subcortical conduction delay along with diffuse conduction delay along the spinal cord. Median SSEPs were abnormal in 30.2% of 43 cases of spinocerebellar ataxia (SCA) patients in a study conducted by Chandran et al. [15]. SSEPs were performed on the right median only, and thus such results could not be interpreted properly.

ATM was the second most common cause, with 25.81% of the total patients; their age ranged from 22 to 35 years, and the mean age was 29 ± 8.9 years with a female-to-male ratio of 1.6:1. All patients had paraparesis, uncontrolled sphincters and sensory level loss with acute onset of symptoms of less than three weeks. Spine MRI showed WMA in three patients; two of them had a hyperintense lesion on T2W images involving multi-segments that occupied the central area on the cross-section and one patient had a hyperintense signal eccentric in location, while the other five patients had normal MRI results. Tibial SSEPs displayed Ab/PWF in five patients of ATM, particularly those who had severe mJOA myelopathy, while median SSEPs were normal in all patients since the lesion level was below cervical spine segments.

Tibial SSEPs were abnormal in 77% of patients with ATM in a study conducted by Kalita and Misra; the median SSEP was abnormal in 15% of patients [16]. Since ATM lesions in our investigation were either in dorsal or lumbar spine segments and no cervical lesion was indicated, explaining the normal median SSEPs in all patients of our study, variability in SSEP outcomes between our study and theirs might be related to the amount of ATM lesions in spinal cord segments. Patients who came with varying clinical presentations of lower limb weakness, incontinence, and vision blurring revealed multiple sclerosis as the third most common cause in 5 (16.13%). One patient was diagnosed with neuromyelitis optica with an abnormal visual evoked potentials (VEP) test bilaterally. Only one patient exhibited abnormal-appearing white matter with a hyperintense T2 signal in the spine MRI of the two patients who had previously experienced remission attacks in the brain. All patients had abnormal SSEPs, either median or tibial or both, and all five patients were in the moderate category based on the mJOA score. Djuric et al. proposed tibial SSEP as a useful criterion for the diagnosis of MS, as it produced abnormal results and the highest sensitivity in MS patients, especially in relapsing-remitting courses [17]. Another study by Crnosija et al. showed that SSEP abnormalities are present in up to 90% patients with definite multiple sclerosis, and in approximately 50% of MS patients without current sensory signs or symptoms. Lower limb (e.g., tibial) SSEPs are more likely to be abnormal than upper limb (e.g., median) SSEPs. However, both upper and lower limb SSEP testing is often indicated because patients may demonstrate abnormalities in only one of these regions [18].

Regarding the subacute combined degeneration of the spinal cord due to vitamin B12 deficiency, NCS revealed sensory impairment in the lower limbs (but did not affect PF latency), and tibial SSEPs displayed delayed P31 and P37 absolute latencies and interpeak latencies (myeloneuropathy) while spine MRI was normal in both patients with vitamin B12 deficiency. In a similar study of 40 patients with vitamin B12 deficiency, MRI revealed an abnormality in 12.5% cases and SSEP abnormality was observed in almost all patients [19]. The unknown cases (patients with idiopathic cause for myelopathy) in this study also had normal spine MRI and NCS but abnormal tibial SSEPs, mainly in dorsal spine segments. An MRI study in patients with ischemia of the anterior spinal artery showed a T2 hyperintense signal within the central gray matter of one patient and in another patient was inconclusive, but tibial SSEPs showed delayed P31 and IPL N22-P31 that signified focal conduction delay at dorsal spine segments and was consistent with the clinical examination of patients. Some studies showed that MRI is normal in up to one-third of patients, especially if done within hours of the event [20].

Our research also had a number of limitations. This was a retrospective research that was conducted at a single centre and had a limited level of information. In order to address this issue, it would be prudent to conduct a study that is prospectively conducted and uses dynamic SSEPs. Also, because not all patients had the same therapy, we were unable to determine whether or not additional factors, such as the impact that various surgical approaches may have on patient outcomes, had an effect. Last, the study had a limited amount of statistical power due to the very small sample size.

## Conclusions

SSEPs had a stronger correlation with the clinical status of cases and their mJOA scores were higher than those for MRI. Furthermore, SSEPs were able to determine cortical involvement for patients with hereditary degenerative ataxias. SSEP measures the functional abnormality of the nervous system regardless of the cause. We recommend performing SSEPs for all patients with non-compressive myelopathy, especially those with negative imaging. Further studies are warranted to validate these findings and to explore the clinical utility of SSEPs in the diagnosis and management of myelopathies.
